# Clinical decision-making on lung cancer investigations in primary care: a vignette study

**DOI:** 10.1136/bmjopen-2023-082495

**Published:** 2024-08-21

**Authors:** Lucy Mitchinson, Christian von Wagner, Alexandra Blyth, Heer Shah, Meena Rafiq, Samuel William David Merriel, Matthew Barclay, Georgios Lyratzopoulos, Willie Hamilton, Gary A Abel, Cristina Renzi

**Affiliations:** 1 Research Department of Behavioural Science and Health, Institute of Epidemiology and Health Care, University College London, London, UK; 2 Department of Primary Care, University of Melbourne, Melbourne, Victoria, Australia; 3 Centre for Primary Care & Health Services Research, The University of Manchester, Manchester, UK; 4 College of Medicine and Health, University of Exeter Medical School, Exeter, UK; 5 Faculty of Medicine, Universita Vita Salute San Raffaele, Milano, Lombardia, Italy

**Keywords:** clinical decision-making, lung diseases, primary care, chronic airways disease, adult oncology

## Abstract

**Abstract:**

**Objectives:**

To investigate the role of comorbid chronic obstructive pulmonary disease (COPD) and symptom type on general practitioners’ (GP’s) symptom attribution and clinical decision-making in relation to lung cancer diagnosis.

**Design:**

Vignette survey with a 2×2 mixed factorial design.

**Setting:**

A nationwide online survey exploring clinical decision-making in primary care.

**Participants:**

109 GPs based in the United Kingdom (UK) who were registered as responders on Dynata (an online survey platform).

**Interventions:**

GPs were presented with four vignettes which described a patient aged 75 with a smoking history presenting with worsening symptoms (either general or respiratory) and with or without a pre-existing diagnosis of COPD.

**Primary and secondary outcome measures:**

GPs indicated the three most likely diagnoses (free-text) and selected four management approaches (20 pre-coded options). Attribution of symptoms to lung cancer and referral for urgent chest X-ray were primary outcomes. Alternative diagnoses and management approaches were explored as secondary outcomes. Multivariable mixed-effects logistic regression was used, including random intercepts for individual GPs.

**Results:**

422 vignettes were completed. There was no evidence for COPD status as a predictor of lung cancer attribution (OR=1.1, 95% CI=0.5–2.4, p=0.914). There was no evidence for COPD status as a predictor of urgent chest X-ray referral (OR=0.6, 95% CI=0.3–1.2, p=0.12) or as a predictor when in combination with symptom type (OR=0.9, 95% CI=0.5–1.8, p=0.767).

**Conclusions:**

Lung cancer was identified as a possible diagnosis for persistent respiratory by only one out of five GPs, irrespective of the patients’ COPD status. Increasing awareness among GPs of the link between COPD and lung cancer may increase the propensity for performing chest X-rays and referral for diagnostic testing for symptomatic patients.

**STRENGTHS AND LIMITATIONS OF THIS STUDY**

- The vignettes were developed using input from patient and public involvement representatives and experienced general practitioners (GPs) to increase validity.
- Use of free-text responses and concealment of the study hypotheses ensured participants were not primed and were able to freely generate their own ideas.
- The vignettes described an ex-smoker—a characteristic that may have alerted GPs and increased lung cancer attributions and urgent chest X-ray requests.
- The vignette scenarios were fairly simple and aiming to elicit a limited number of likely diagnoses and management options due to pragmatic and sample size considerations.

## Introduction

 Chronic obstructive pulmonary disease (COPD) is a lung condition that affects 2% of the United Kingdom (UK) population and is an independent risk factor associated with twice the risk of lung cancer.^1 2^ The main symptoms of COPD are shortness of breath, chronic cough, frequent chest infections and wheezing.^3^ COPD has also been associated with two-times longer intervals before help-seeking for new symptoms.^4^ Lung cancer is the third most common cancer in the UK and the most common cause of cancer death.^5^ Late diagnosis of lung cancer greatly impacts survival, with 1-year net survival dropping from 71.1% when diagnosed at stage 1, to 34.3% for stage 3 and to 15.5% survival for stage 4.^6^ Late cancer diagnosis can be attributed to multiple factors, potentially including the presence of pre-existing chronic conditions (termed comorbidity).^7^ Timely referral for chest X-ray and lung cancer diagnostic pathways are essential for enabling early-stage diagnosis and improving chances of survival.

Clinical decisions such as these are influenced by several factors, including the likelihood of potential diagnoses, patient symptoms and underlying risk.^8^,^10^ Diagnostic delay may be amplified in primary care if general practitioners (GPs) solely attribute lung cancer symptoms to a patient’s pre-existing condition, for instance, shared common symptoms such as chronic cough or dyspnoea in the case of lung cancer (ie, alternative explanation mechanism).^8^,^11^ Additionally, delays might also be due to prioritising management of the current conditions over the investigation of new or worsening symptoms (ie, competing demands mechanism).^8 9^ Attribution of potential lung cancer symptoms to existing or less severe conditions may occur more often for patients with chronic comorbidities and lead to inequalities in cancer investigation, referral and diagnosis.

In 2015, the National Institute for Health and Care Excellence (NICE) lowered the threshold for cancer investigations, from symptom-associated positive predictive value (PPV) of 5–3%, but do not consider the role of comorbidity.^12^ It is necessary to investigate how the presence of COPD can affect symptom attribution and management approaches in general practice when patients present with new or worsening symptoms. GPs may be aware of the link between COPD and lung cancer and refer patients with COPD for urgent X-ray more readily due to the increased cancer risk; however, the alternative explanation mechanism suggests that some GPs may instead attribute the symptoms to COPD and choose to manage the condition rather than refer for an X-ray. Understanding clinical decision-making for lung cancer diagnosis when patients present with chronic conditions associated with increased cancer risk is essential for supporting earlier diagnosis and improving survival.^13^

This study aimed to investigate the role of comorbid COPD on GP’s symptom attribution and clinical decision-making in relation to lung cancer diagnosis.

The research questions are as follows:

1. How does a patient’s COPD status influence a GP’s attribution of worsening respiratory or general symptoms to lung cancer?
2. How does a patient’s COPD status influence a GP’s decision to refer the patient for an urgent chest X-ray?
3. How does a patient’s COPD status in combination with the presentation of respiratory versus general symptoms influence a GP’s decision to refer the patient for an urgent chest X-ray?

The primary outcomes of interest are (1) attribution of symptoms to lung cancer and (2) referral for urgent chest X-ray. These outcomes reflect a rapid response to potential lung cancer symptoms and are in line with the current NICE guidelines. The secondary outcomes of interest are (3) attribution of symptoms to alternative diagnoses and (4) selection of other management approaches. These outcomes will provide insight into alternative explanations attributed to patients, and how COPD status and attributed diagnoses may influence GP’s management responses.

## Methods

### Design

A 2×2 mixed factorial design vignette survey was conducted, with vignettes describing patients with either worsening respiratory symptoms or worsening general symptoms, as well as with or without a pre-existing COPD diagnosis. Vignettes are short-text descriptions of a situation which permit the manipulation of specific symptoms within a fixed context.^14^ Vignettes have been successfully used for understanding the processes of clinical practice and examining which factors influence diagnostic processes when patients have complex needs.^14^,^17^

The online survey presented four vignettes in a randomised order, which described a hypothetical male patient. The patient was aged 75 with a history of smoking and presenting with either respiratory symptoms (dyspnoea and persistent cough) or general symptoms (fatigue and weight loss) which have been worsening for the past 3 weeks. These symptoms are independently associated with lung cancer with PPV above 3% (the normative threshold used by NICE to justify urgent referral recommendations), and their presence warrants an urgent chest X-ray according to NICE guidelines.^12 18 19^ The patient also had either a prior COPD diagnosis or no COPD diagnosis. The vignettes included clinical information, such as details on smoking history and ethnicity (vignettes presented in online supplemental material). The vignette design was informed by previous studies.^11 20^

### Patient and public involvement

Patient and public involvement (PPI) representatives and GPs reviewed the proposed vignettes and provided constructive feedback and insight during vignette development. Minor improvements were made to vignettes in response to their feedback. Members of the SPOCC (SPOtting Cancer among Comorbidities) research PPI group also contributed to steering group meetings and were updated on emerging findings.

### Outcome measures

#### Symptom attribution

To explore how a patient’s COPD status influences a GP’s attribution of worsening respiratory or general symptoms to lung cancer (research question 1), GPs were asked: ‘What do you think are the three most likely diagnoses, starting from the most likely to the least likely?’ after reading each vignette. The free-text responses were analysed and coded into categories developed during content analysis (eg, ‘lung cancer’ or ‘respiratory infections’) to form the measure of ‘symptom attribution’.^17^

To explore the primary attribution outcome (eg, attribution of symptoms to lung cancer), the responses were dichotomised to ‘lung cancer’ or ‘not lung cancer’ for analyses. To explore the secondary attribution outcome (eg, attribution of symptoms to alternative diagnoses), the initial attribution categories were used.

#### Management approaches

To explore whether a patient’s COPD status influences a GP’s decision to refer the patient for an urgent chest X-ray (research question 2), and whether this changed when presenting in combination with respiratory versus general symptoms (research question 3), participants were asked to choose up to four management approaches from a list of 20 pre-coded options. The approaches were ranked by priority, with ‘1’ being the ‘highest priority’ and ‘4’ being the ‘lowest priority’ to form the measure of management approach.

To explore the primary management outcome (eg, referral for urgent chest X-ray), responses were dichotomised to ‘urgent chest X-ray’ or ‘not urgent chest X-ray’. Only the highest priority approach (out of four possible responses) was considered for this analysis, as delays might occur if urgent chest X-ray was not the top priority. To explore the secondary management outcome (eg, selection of other management approaches by GPs), all management approaches were used in the analysis.

Participant’s demographic data were collected via pre-coded categorical responses, including GP gender, years of GP experience and GP practice size. These variables were included as potential covariates in the analyses.

### Participants

Participants were UK GPs who were registered as responders on the Dynata survey platform (www.dynata.com). Participants who met the criteria of (1) being based in the UK and (2) being qualified GPs were recruited in May and June 2022 via a Dynata advertisement. No additional inclusion or exclusion criteria were applied. GPs were compensated £38.50 for completing the survey. Participants whose survey completion times were deemed too short were removed for quality assurance purposes (eg, <3 min).

Consenting participants were asked to complete, as a minimum, two of the four vignettes (providing 200–400 completed vignettes). The sample size calculation indicated that a sample of 100 GPs was required to attain 194 completed vignettes to detect a 20% difference in odds of GPs attributing symptoms to lung cancer when seeing patients with COPD versus without, at a significance level of p<0.05 and 80% power.

### Statistical analysis

#### Symptom attribution

A three-way cross-tabulation was used to examine the frequency of GP that suggested diagnoses by scenario. Logistic univariable regression analyses were used to compare the frequency of suggested diagnosis categories within each symptom by COPD status.

The primary outcome of interest (eg, attribution of symptoms to lung cancer) was analysed using two models:

1. ‘Most likely’ model: GPs considered lung cancer as the first or most likely cause for symptoms.
2. ‘Any’ model: GPs suggested lung cancer as either the first, second or third potential cause of the symptoms.

Attributing symptoms to lung cancer as the ‘most likely’ cause should lead to the smallest diagnostic delay. Attributing symptoms to lung cancer at ‘any’ likelihood would lead to investigations but some delay might occur.

Multivariable mixed-effects logistic regression was used to examine the associations between vignette scenario and attribution to lung cancer, adjusting for GP characteristics.

#### Management approaches

Using a three-way cross-tabulation we examined the frequency of the highest priority management approaches by scenario. Logistic univariable regression analyses were used to compare the frequency of selected management approaches within each symptom by COPD status.

The primary outcome of interest was referral for urgent chest X-ray as the top priority approach. Logistic univariable mixed-effects logistic regression was used to explore the associations between urgent chest X-ray referral and vignette scenario. Multivariable mixed-effects logistic regression was conducted to adjust for GP characteristics and to include lung cancer attribution as a covariate.

A random intercept was employed for each GP to account for repeated observations. The reference group for analyses was respiratory symptom and no COPD history. All analyses were completed using StataMP V.17. Incomplete responses (eg, missing ‘most likely’ diagnosis or ‘top’ priority action) were excluded. ORs, 95% CIs and p values for all analyses are presented as tables and forest plots in the supplemental material.

## Results

A total of 422 vignettes were completed by 109 GPs. In all, 93% of GPs completed all four vignettes. There were comparable numbers of male (n=55) and female (n=54) participants (national average is 49.5% female).^21^ Most GPs had 20+ years of experience (40%). Over half the sample worked in practices with more than five other doctors (58%) (table 1). The Business Services Authority reported that in April 2024 in England, 50% of practices reported having more than five GPs, 32% reported four to five GPs, 6% reported one to two GPs and 12% of practices had a single GP.^22^ Experience groups were dichotomised (≤10 and >10 years) and solo practicing GPs were combined with those working with two to three others for further analyses.

**Table 1:** Sample characteristics

Characteristics	N (column %)	
Gender		
Male	55	(50.5)
Female	54	(49.5)
Total	109	
Experience (years)		
0–5	5	(4)
6–10	18	(17)
11–15	25	(23)
16–20	17	(16)
20+	44	(40)
Total	109	
Practice size		
Single GP practice	2	(2)
2–3 GPs	13	(12)
4–5 GPs	31	(28)
5+ GPs	63	(58)
Total	109	

GP, general practitioner

### Symptom attribution

#### Primary outcome: attribution of symptoms to lung cancer

**Figure 1:**
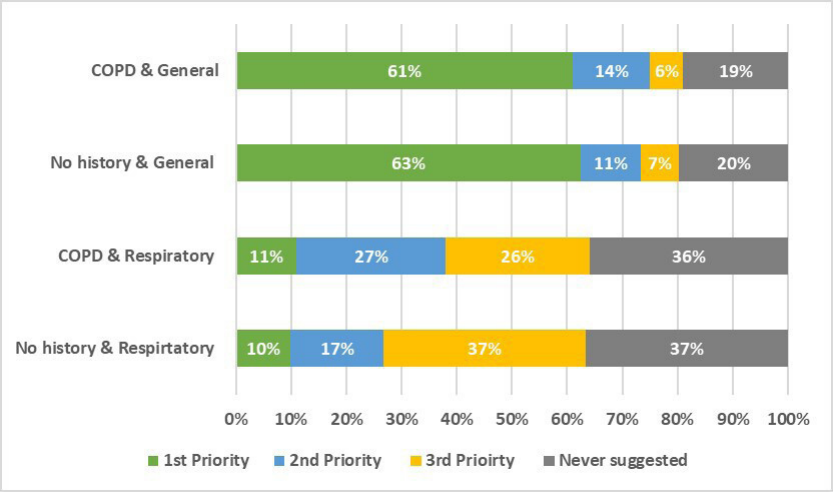
Percentage of lung cancer attributions made at each level of likelihood (ie, suggested as the first, second or third most likely diagnosis or never suggested) for each vignette scenario. GPs were asked to suggest three potential diagnoses for each vignette ranked from most to least likely. COPD, chronic obstructive pulmonary disease; GP, general practitioner.

Lung cancer was suggested as the ‘most likely’ cause in 11% of patients with respiratory symptoms and COPD, and 10% of patients with respiratory symptoms and no COPD (table 2, figure 1). When considering the top three possible diagnoses (rather than only the ‘most likely’ diagnosis), lung cancer was suggested in 21% of responses for both patients with respiratory symptoms and COPD, and for patients with respiratory symptoms and no COPD (table 2).

**Table 2:** Frequency of overall and most likely symptom attributions by COPD status and presenting symptom

	Symptom attribution	Most likely symptom attribution					Overall symptom attribution				
		No COPDn (column %)		COPDn (column %)		Univariable regression p value	No COPDn (column %)		COPDn (column %)		Univariable regression p value
Worsening respiratory symptoms (dyspnoea+persistent cough)	Lung cancer	10	(10)	12	(11)	0.594	66	(21)	67	(21)	0.914
	Other cancers (excluding lung cancer)	1	(1)	1	(1)	*	7	(2)	10	(3)	0.297
	**COPD (and/or exacerbation /mismanagement**)	**21**	(**20**)	**51**	(**49**)	**0.001**	**32**	(**10**)	**66**	(**21**)	**<0.001**
	Respiratory infection	13	(13)	11	(11)	0.57	43	(14)	38	(12)	0.326
	**Other respiratory conditions**	**49**	(**47**)	**22**	(**21**)	**<0.001**	**84**	(**27**)	**52**	(**17**)	**<0.001**
	Cardiac conditions	9	(9)	8	(8)	0.73	72	(23)	75	(24)	0.623
	Other conditions (excluding respiratory and cardiac conditions)	0		0		*	4	(1)	4	(1)	*
	Patient factors	1	(1)	0		*	2	(1)	2	(1)	*
	Total	104		105			310		314		
Worsening general symptoms (fatigue+weight loss)	Lung cancer	67	(63)	65	(61)	0.813	86	(27)	86	(27)	0.692
	Other cancers (excluding lung cancer)	15	(14)	15	(14)	0.707	51	(16)	41	(13)	0.086
	**COPD (and/or exacerbation /mismanagement**)	1	(1)	6	(6)	*	**18**	(**6**)	**42**	(**13**)	**<0.001**
	Respiratory infection	3	(3)	3	(3)	*	17	(5)	20	(6)	0.443
	Other respiratory conditions	17	(16)	12	(11)	0.275	**65**	(**20**)	**46**	(**15**)	**<0.001**
	Cardiac conditions	1	(1)	3	(3)	*	46	(14)	50	(16)	0.34
	Other conditions (excluding respiratory and cardiac conditions)	2	(2)	1	(1)	*	35	(11)	30	(9)	0.378
	Patient factors	1	(1)	1	(1)	*	1	(1)	1	(1)	*
	Total	107		106			321		318		

GPs were asked to suggest three potential diagnoses for each vignette ranked from most to least likely

Bold indicates significance at the p<0.05 level.

* Comparisons only reported where cell values are greater than 5. Bold indicates significance at the p level. COPD: , GP: General Practitioner.

COPDchronic obstructive pulmonary diseaseGPgeneral practitioner

Lung cancer was suggested as the ‘most likely’ cause in 61% of patients with general symptoms and COPD, and 63% of patients with general symptoms and no COPD (table 2). When considering the top three possible diagnoses, lung cancer was suggested in 27% of responses for both patients with general symptoms and COPD, and for patients with general symptoms and no COPD (table 2).

#### Research question 1: how does a patient’s COPD status influence a GP’s attribution of worsening respiratory or general symptoms to lung cancer?

In univariable analysis, there was strong evidence for general symptoms being associated with a higher probability of lung cancer attribution than respiratory symptoms (OR=6.4; 95% CI=2.4–17.3, p<0.001, online supplemental table 1). This was confirmed in multivariable analysis, with general versus respiratory symptoms being associated with lung cancer attribution independently of the COPD status when controlling for GP characteristics (OR=6.4, 95% CI=2.4–17.2, p<0.001, online supplemental table 2 and figure 1). There was no evidence for COPD status as a predictor of lung cancer attribution (OR=1.1, 95% CI=0.5–2.4, p=0.914). Findings were similar when considering ‘most likely’ diagnosis in univariable and multivariable analyses (online supplemental tables 3, 4 and figure 1).

#### Secondary outcome: attribution of symptoms to alternative explanations

For patients with worsening respiratory symptoms and COPD, the diagnoses most frequently suggested as the ‘most likely’ cause were exacerbations of COPD (suggested by 49% of GPs) followed by other respiratory conditions (suggested by 21% of GPs). The opposite was seen for patients with respiratory symptoms and no COPD, as ‘other respiratory conditions’ was the most frequently suggested cause (suggested by 47% of GPs) followed by undiagnosed COPD (suggested by 20% of GPs) (table 2).

### Management approaches

#### Primary outcome: referral for urgent chest X-ray

##### Research question 2: how does a patient’s COPD status influence a GP’s decision to refer the patient for an urgent chest X-ray?

Patients with respiratory symptoms and COPD had slightly lower odds of urgent chest X-ray referral than patients with respiratory symptoms and no COPD in univariable (OR=0.6, 95% CI=0.3–1.1, p=0.117, online supplemental table 5) and multivariable analyses (OR=0.6, 95% CI=0.3–1.2, p=0.12, online supplemental table 6) without reaching statistical significance.

There was some evidence that lung cancer attribution was associated with a higher probability of urgent chest X-ray referral when included in the multivariable model (OR=2.5; 95% CI=1.2–5.1, p=0.011, online supplemental table 7). This was confirmed in multivariable analysis, where suggestion of lung cancer as the ‘most likely’ diagnosis was associated with a higher probability of urgent chest X-ray referral (OR=3.0, 95% CI=1.5–6.2, p=0.003, online supplemental table 8).

##### Research question 3: how does a patient’s COPD status in combination with the presentation of respiratory versus general symptoms influence a GP’s decision to refer the patient for an urgent chest X-ray?

Patients with general symptoms and COPD had slightly lower odds of urgent chest X-ray referral than patients with respiratory symptoms and no COPD in univariable (OR=0.9, 95% CI=0.5–1.7, p=0.744, online supplemental table 5) and multivariable analyses (OR=0.9, 95% CI=0.5–1.8, p=0.767, online supplemental table 6) without reaching statistical significance.

### Secondary outcome: selection of other management approaches

**Figure 2:**
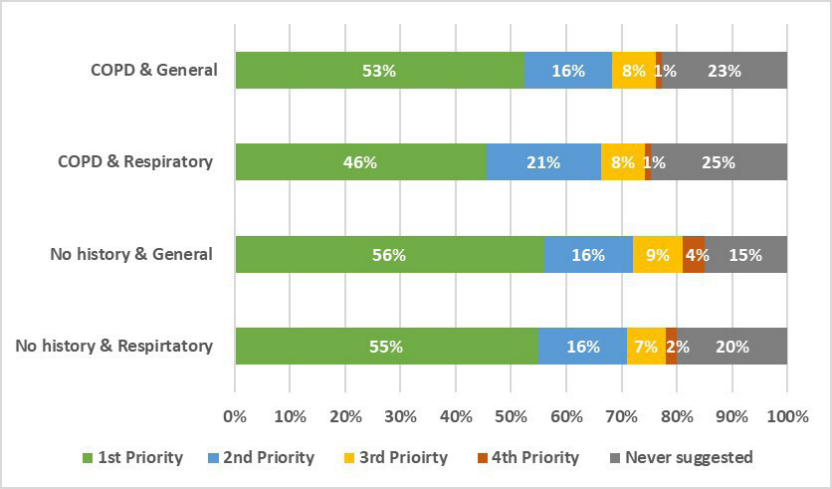
Proportion of urgent chest X-ray referrals selected at each level of priority (ie, selected as the first, second, third or fourth priority management approach) for each vignette scenario. COPD, chronic obstructive pulmonary disease.

Urgent chest X-ray was the most frequently selected approach for all four vignettes (46–56% of responses, figure 2). The next most frequently selected approach was ‘prescribing or changing medication’ for patients with worsening respiratory symptoms and COPD (22%) and for patients with worsening respiratory symptoms and no COPD (15%) (table 3). For patients with general symptoms, ‘blood tests’ were the next most frequently chosen approach, selected for 19% of patients with worsening general symptoms and COPD, and for 21% of patients with worsening general symptoms and no COPD.

**Table 3:** Frequency of top priority management approach and all management approaches by COPD and presenting symptoms

	Management approaches	Top priority approach					All approaches				
		No COPDn (column %)		COPDn (column %)		Univariable regressionp value	No COPDn (column %)		COPDn (column %)		Univariable regressionp value
Worsening respiratory symptoms(dyspnoea+persistent cough)	Urgent chest X-ray	57	(55)	48	(46)	0.117	83	(21)	79	(20)	0.373
	Non-urgent chest X-ray	6	(6)	1	(1)	*	16	(4)	17	(4)	0.877
	**Prescribe or change medications and book an appointment for review within a few days**	16	(15)	23	(22)	0.098	**31**	(**8**)	**46**	(**12**)	**0.017**
	Blood tests (eg, FBC panel, U&E, LFT, CRP or BNP) or sputum culture	12	(12)	19	(18)	0.099	149	(38)	140	(36)	0.505
	Refer to respiratory specialist, COPD nurse, pulmonary rehabilitation programme, spirometry, gastroenterologist or cardiologist	9	(9)	7	(7)	0.541	67	(17)	63	(16)	0.679
	CT scan or ECG	3	(3)	3	(3)	*	26	(7)	25	(6)	0.906
	Refer to A&E	1	(1)	3	(3)	*	6	(2)	8	(2)	0.468
	Patient monitor symptoms and return if they worsen or change	0		1	(1)	*	9	(2)	8	(2)	0.678
	Thyroid function or HbA1c	0		0		*	5	(1)	6	(2)	0.76
	Total	104		105			392		392		
Worsening general symptoms (fatigue+weight loss)	Urgent chest X-ray	60	(56)	56	(53)	0.619	91	(23)	82	(21)	0.121
	Non-urgent chest X-ray	1	(1)	2	(2)	*	8	(2)	4	(1)	*
	Prescribe or change medications and book an appointment for review within a few days	2	(2)	2	(2)	*	13	(3)	20	(5)	0.128
	Blood tests (eg, FBC panel, U&E, LFT, CRP or BNP) or sputum culture	22	(21)	20	(19)	0.589	142	(35)	141	(36)	0.934
	Refer to respiratory specialist, COPD nurse, pulmonary rehabilitation programme, spirometry, gastroenterologist, cardiologist or endoscopy	12	(11)	11	(11)	0.779	68	(17)	67	(17)	0.97
	CT scan or ECG	8	(8)	12	(11)	0.226	44	(11)	48	(12)	0.611
	Refer to A&E	2	(2)	3	(3)	*	7	(2)	7	(2)	0.997
	Patient monitor symptoms and return if they worsen or change	0		0		*	4	(1)	1	(0)	*
	Thyroid function or HbA1c	0		0		*	26	(6)	27	(7)	0.864
	Total	107		106			403		397		

Frequencies for individual management approaches presented in online supplemental table 9.

* Comparisons only reported where cell values are greater than 5. Bold indicates significance at the pp<0.05 level.

A&E, accident and emergency; BNP, B-type natriuretic peptide; COPD, chronic obstructive pulmonary disease; CRP, C-reactive protein; CT, Computerised Tomography; ECG, Electrocardiogram; FBC, full blood count; GP, general practitioner; hba1c, haemoglobin A1Conline supplemental table 9)HbA1chaemoglobin A1cLFT, liver function test; U&E, urea and electrolytes

The selection of management approach varied with the ‘most likely’ attribution. Urgent chest X-ray was the top priority approach for respiratory symptoms when the ‘most likely’ attribution was lung cancer (68%) or other respiratory conditions (58%) (table 4). Urgent chest X-ray was the top priority approach for general symptoms when the ‘most likely’ attribution was lung cancer (62%), exacerbations of COPD (57%) or other respiratory conditions (55%) (table 4).

**Table 4:** Frequency of single highest priority management approach by ‘most likely’ diagnosis and presenting symptoms

	Top priority management approach	Most likely symptom attribution										
		Lung cancern (column %)		COPDn (column %)		Respiratory conditionn (column %)		Cardiac conditionn (column %)		Other condition n (column %)		Univariable regression (p value)
Worsening respiratory symptoms (dyspnoea+persistent cough)	Urgent chest X-ray	15	(68)	30	(42)	41	(58)	7	(41)	12	(44)	0.689
	Non-urgent chest X-ray	0		1	(1)	3	(4)	0		3	(11)	0.132
	Blood tests (eg, FBC panel, U&E, LFT, CRP or BNP) or sputum culture	2	(9)	8	(11)	8	(11)	8	(47)	5	(19)	0.132
	Prescribe or change medications and review within a few days	0	(9)	31	(43)	2	(3)	0		6	(22)	0.113
	Referral to respiratory specialist, COPD nurse, pulmonary rehabilitation programme or spirometry	2	(9)	1	(1)	13	(18)	0		0		0.89
	CT scan or ECG	1	(5)	0		3	(4)	2	(12)	0		0.642
	Refer to A&E	2	(9)	0		1	(1)	0		1	(4)	*
	Monitor symptoms	0		1	(1)	0		0		0		*
	Total	22		72		71		17		27		
Worsening general symptoms (fatigue+weight loss)	Urgent chest X-ray	82	(62)	4	(57)	16	(55)	1	(25)	13	(32)	0.005
	Non-urgent chest x-ray	3	(2)	0		0		0		0		*
	**Blood tests (eg, FBC panel, U&E, LFT, CRP**)	**14**	(**11**)	**0**		**5**	(**17**)	**2**	(**50**)	**21**	(**51**)	**0.003**
	Prescribe or change medications and review within a few days	0		2	(29)	0		0		2	(5)	*
	Referral to respiratory specialist, COPD nurse, spirometry or gastroenterologist	17	(13)	0		2	(7)	0		4	(10)	0.244
	CT scan or ECG	13	(10)	1	(14)	4	(14)	1	(25)	1	(2)	0.506
	Refer to A&E	3	(2)	0		2	(7)	0		0		*
	Total	132		7		29		4		41		

Bold indicates significance at the p<0.05 level.

* Comparisons only reported where row values are greater than 5. Bold indicates significance at the p level.

A&E, accident and emergencyBNP, B-type natriuretic peptide; COPD, chronic obstructive pulmonary disease; CRP, C-reactive protein; ECG, Electrocardiogram; FBC, full blood count; GP, general practitioner; LFT, liver function test; scan, CT; U&E, urea and electrolytes

If GPs felt COPD was the ‘most likely’ cause, then GPs chose to ‘prescribe or change medications and book to review’ (43%) most frequently. There was no evidence for an association between COPD status and selection of the top priority management approach for either symptom type (table 3, urgent chest X-ray referral for respiratory symptoms, p=0.117, and general symptoms, p=0.619).

## Discussion

Lung cancer was suggested as a possible cause of worsening respiratory symptoms by one-fifth of GPs participating in this study. GP responses on lung cancer attribution did not differ by patient’s COPD status, despite the greater risk of developing cancer for patients with COPD. GPs most frequently attributed worsening symptoms to COPD for patients with the chronic condition or to other respiratory diseases for patients without COPD. Worsening general symptoms were attributed to lung cancer more frequently than respiratory symptoms. The management approaches selected by GPs depended on the diagnoses they considered to be most likely, with lung cancer attribution associated with a higher probability of urgent chest X-ray referral. While overall this was the most frequently selected approach, in the case of patients with worsening respiratory symptoms attributed to exacerbations of COPD, GPs more often prescribed medications.

The first diagnostic impressions of healthcare professionals are important and have been strongly associated with clinical decision-making and cancer diagnosis.^23^ The worsening respiratory symptoms included in this study are regarded as typical lung cancer alarm symptoms in NICE guidelines, particularly for older patients with a history of smoking but regardless of the COPD status.^12^ In a relatively large proportion of responses, GPs did not mention lung cancer among the three most likely causes and most frequently attributed the worsening respiratory symptoms to exacerbation or mismanagement of COPD or other respiratory conditions. While respiratory symptoms such as these are seen in COPD, the duration should raise concerns as it captures the persistent and worsening nature of the symptoms.

Lung cancer may be perceived as more likely if symptoms persist for longer, such as over 3 months; however, delaying investigation in patients with comorbid COPD may lead to later stage diagnosis and poorer outcomes. Another vignette study that did not include the presence of comorbidities found that GP symptom investigation was not influenced by the presence of high or low cancer-risk symptoms.^20^ While this might be justified by the relative low frequency of lung cancer in the general population compared with other conditions, not considering a potential lung cancer diagnosis was associated with a lower likelihood of urgent referrals for investigations, increasing the risk of diagnostic delays.

Urgent chest X-ray referral was the most common management approach for GPs in this study, although this choice did not appear to be influenced by the COPD status. There is growing primary care evidence for the role chest X-ray plays in lung cancer diagnosis, with some evidence that increased use of chest X-ray leads to stage shift in lung cancer diagnoses.^24^ Diagnostic tests should be conducted more frequently for patients with comorbidities who present with new/worsening symptoms to rule out cancer as a possibility. Substantial variation in chest X-ray usage exists at GP practice level which, if addressed, could reduce geographical and region differences in lung cancer outcomes.^25^ Future studies may explore whether GPs would select to monitor symptoms in return appointments after selecting certain management approaches (such as adjusting medications) to allow for in-depth exploration of GP decision-making and the influence of the symptom period (eg, experiencing symptoms for 3 months compared with 3 weeks).

NICE guidance recommends that persistent respiratory symptoms, experienced for 3 weeks or more, should prompt GP attendance and further investigation.^12^ Despite public health campaigns raising awareness of the potential significance of persistent respiratory symptoms for undiagnosed lung cancer such as ‘Be Clear on Cancer’,^26^ patients with comorbidities may delay help-seeking due to symptom attribution to their pre-existing conditions. A previous vignette study showed only 20% of patients attributed new respiratory symptoms (dyspnoea and persistent cough) to lung cancer and patients with existing respiratory conditions were more likely to attribute respiratory symptoms to asthma or COPD.^17^ GPs should potentially have a higher index of suspicion for lung cancer in patients with comorbidities as patients may have already attributed symptoms to an alternative explanation and delayed presentation to primary care.^4^ Use of decision support tools which incorporate comorbidities (such as COPD) and additional electronic health record data could be used in clinical practice to support this. Patients who are at a higher risk of underlying lung cancer could be identified using the tools and prioritised for urgent referrals, particularly when presenting with new or worsening symptoms or if they do not respond to initial drug treatments.

It should be noted that evidence on the role of comorbidities (such as COPD) in influencing stage at lung cancer diagnosis is mixed, with previous studies suggesting possible protective effects.^27 28^ A recent study of over 86 000 patients from Ontario, Canada, with lung cancer diagnosed between 2008 and 2020 showed a 30% lower risk of late-stage diagnosis for patients with COPD compared with those without. This effect was only slightly tempered by previous chest imaging.^29^ Patients with chronic conditions, such as COPD, have more frequent contact with healthcare services for disease monitoring which may result in earlier cancer detection. Therefore, other mechanisms may in practice mitigate the observed similarity in outcome by the COPD status. Particular attention should be dedicated to patients with chronic conditions who do not engage with healthcare as these patients are at high risk of both poor health outcomes from their chronic condition and delays in lung cancer diagnosis.

Vignette studies are an effective tool for exploring the psychological mechanisms involved in clinical decision-making.^14^,^17^ The current study provided new insights into how GPs interpret symptoms and how the presence of pre-existing conditions may influence diagnosis. The vignettes were developed using input from PPI representatives and experienced GPs to increase validity. The completion of four vignettes by 93% of participants indicates high acceptability and feasibility. Use of free-text responses and concealment of the study hypotheses ensured participants were not primed and were able to freely generate their own ideas.

There are also some limitations to this work. First, the vignettes described an ex-smoker and this characteristic was not varied between the cases presented to GPs. Smoking status may affect GP’s symptom attribution and decision-making, and future vignette studies should consider varying such risk factors to evaluate their possible role in influencing clinical decision-making. Additionally, for practical reasons and sample size considerations, the vignette scenarios were fairly simple, aiming to elicit the three most likely diagnoses and up to four preferred management options. Often GPs will appropriately suspect even more possible diagnoses or consider more than four actions (at times in combination with a referral). Lastly, GPs were recruited via an online survey platform with limited inclusion criteria. While this allowed for wide participation of UK GPs, our sample was not directly representative of the UK. We had a greater proportion of GPs who worked in larger practices. There is a potential that GPs from larger practices experience more patients with lung cancer or have better systems in place for lung cancer referral.

There is currently limited evidence on the role of comorbidity in GP’s decision-making regarding cancer investigations. This study begins to address this by exploring the effect of COPD morbidity on cancer symptom attribution and diagnostic investigations in primary care. COPD was not a clear factor in influencing GP’s decision-making on diagnostic investigations or cancer risk perception when patients presented with possible cancer symptoms and pre-existing morbidity. Further research identifying the clinical features associated with underlying lung cancer in patients with COPD is needed to inform updates to the NICE guidelines. The provision of integrated healthcare is particularly important as doctor-patient interactions for managing chronic conditions can offer an opportunity for early cancer detection and diagnosis. This is especially important in the context of an ageing population, with increasing number of people with multimorbidity.

Despite emerging evidence about possible protective effects of comorbidities on cancer stage at diagnosis, there is still scope for increasing GP clinical suspicion of lung cancer among patients with COPD and use of prompt diagnostic investigations for patients with COPD who have worsening symptoms.

## supplementary material

10.1136/bmjopen-2023-082495 online supplemental file 1

## Data Availability

Data are available upon reasonable request.
