# Supplementary material for: Clinical decision-making on lung cancer investigations in primary care: a vignette study
Source: BMJ Open. 2024 Aug 21;14(8):e082495. doi: 10.1136/bmjopen-2023-082495 (PMC11340710; doi:10.1136/bmjopen-2023-082495)
Supplement: online supplemental file 1 [file bmjopen-14-8-s001.pdf]

Supplementary material: Clinical decision-making on lung cancer investigations in primary care: a vignette study

GP-facing vignettes

<u>Respiratory symptoms</u>

Vignette 1 (COPD with shortness of breath and cough)

- A white, male patient, aged 75 years, is sitting in front of you.
- He has a diagnosis of COPD, and is an ex-smoker, with a history of 25 pack-years.
- He has been experiencing worsening shortness of breath after exertion, like walking uphill, and a persistent cough with white sputum, for the past three weeks.
- He finds that these symptoms have made it difficult to carry out activities he could perform before, such as performing household chores, as well as activities he enjoyed, such as going for a short walk in the park.
- The patient shows no signs of finger clubbing.
- His chest is clear on auscultation, though his peak flow has reduced, with the FEV1 at 75% of expected.
- His temperature is normal, heart rate is 90 beats per minute. His respiratory rate is 18 breaths per minute and oxygen saturation on air is 94%.
- There are no other symptoms of note.
- The patient regularly attends his routine, annual health check-ups.

Vignette 2 (No COPD with shortness of breath and cough)

- A white, male patient, aged 75 years, is sitting in front of you.
- He is an ex-smoker, with a history of 25 pack-years.
- He has been experiencing worsening shortness of breath after exertion, like walking uphill, and a persistent cough with white sputum, for the past three weeks.
- He finds that these symptoms have made it difficult to carry out activities he could perform before, such as performing household chores, as well as activities he enjoyed, such as going for a short walk in the park.
- The patient shows no signs of finger clubbing.
- His temperature is normal, heart rate is 90 beats per minute. His respiratory rate is 18. breaths per minute and oxygen saturation on air is 94%.
- There are no other symptoms of note.
- The patient regularly attends his routine, annual health check-ups.

<u>General symptoms</u>

Vignette 3 (COPD with fatigue and weight loss)

- A white, male patient, aged 75 years, is sitting in front of you.
- He has a diagnosis of COPD, and is an ex-smoker, with a history of 25 pack-years.
- He has been experiencing worsening fatigue and a significant loss of weight, for the past three weeks.
- He finds that these symptoms have made it difficult to carry out activities he could perform before, such as performing household chores, as well as activities he enjoyed, such as going for a short walk in the park.
- The patient shows no signs of finger clubbing.
- His chest is clear on auscultation, though his peak flow has reduced, with the FEV1 at 75% of expected.

- His temperature is normal, heart rate is 90 beats per minute. His respiratory rate is 18 breaths per minute and oxygen saturation on air is 94%.
- There are no other symptoms of note.
- The patient regularly attends his routine, annual health check-ups.

Vignette 4 (No COPD with fatigue and weight loss)

- A white, male patient, aged 75 years, is sitting in front of you.
- He is an ex-smoker, with a history of 25 pack-years.
- He has been experiencing worsening fatigue and a significant loss of weight, for the past three weeks.
- He finds that these symptoms have made it difficult to carry out activities he could perform before, such as performing household chores, as well as activities he enjoyed, such as going for a short walk in the park.
- The patient shows no signs of finger clubbing.
- His temperature is normal, heart rate is 90 beats per minute. His respiratory rate is 18 breaths per minute and oxygen saturation on air is 94%.
- There are no other symptoms of note.
- The patient regularly attends his routine, annual health check-ups.

Supplementary material: Clinical decision-making on lung cancer investigations in primary care: a vignette study

Symptom attribution analysis

Table S.1 Odds ratio, 95% CIs and p values for 'overall' level of symptom attribution (i.e., of ever mentioning lung cancer) from the mixed-effects logistic regression with a random intercept for each individual GP.

	Overall			
**Predictor**	**OR**	**95% CI**		**P**
		**Lower**	**Upper**	
**Unadjusted**				
Vignette (COPD & General vs No History & Respiratory)	***7.86***	***2.81***	***22.02***	***<0.001***
Vignette (No History & General vs No History & Respiratory)	***6.42***	***2.38***	***17.34***	***<0.001***
Vignette (COPD & Respiratory vs No History & Respiratory)	*1.05*	*0.45*	*2.42*	*0.914*

Table S.2 Odds ratio, 95% CIs and p values for 'overall' level of symptom attribution (i.e., of ever mentioning lung cancer) from the mixed-effects logistic regression, adjusting for gender, GP experience, number of co-workers and with a random intercept for each individual GP.

	Overall			
**Predictor**	**OR**	**95% CI**		**P**
		**Lower**	**Upper**	
**Adjusted for GP Gender, Experience and Number of Co-workers**				
Vignette (COPD & General vs No History & Respiratory)	***7.84***	***2.8***	***21.92***	***<0.001***
Vignette (No History & General vs No History & Respiratory)	***6.38***	***2.37***	***17.2***	***<0.001***
Vignette (COPD & Respiratory vs No History & Respiratory)	*1.05*	*0.45*	*2.41*	*0.914*
Gender (Male vs Female)	*1.97*	*0.48*	*8.07*	*0.346*
GP Experience (11-20 vs 1-10 years)	*4.52*	*0.64*	*31.97*	*0.131*
GP Experience (>20 vs 1-10 years)	*5.53*	*0.82*	*37.44*	*0.08*
GP Co-workers (4-5 vs Solo-3)	*1.51*	*0.16*	*14.5*	*0.724*
GP Co-workers (>5 vs Solo-3)	*2.68*	*0.36*	*20.18*	*0.339*

Note: Bold = p<0.05

Supplementary material: Clinical decision-making on lung cancer investigations in primary care: a vignette study

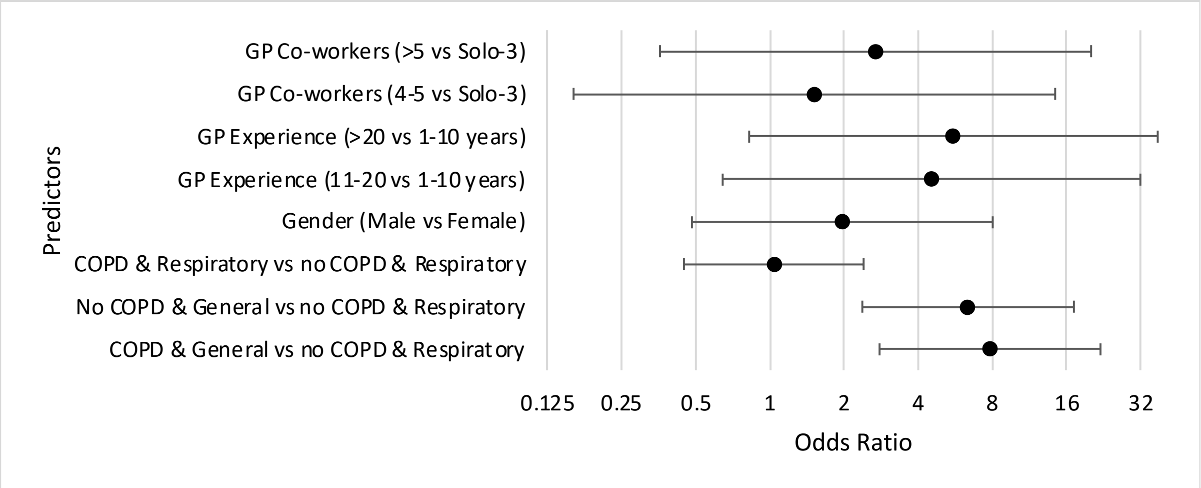

Figure S.1 Odds ratio and 95% CIs for 'overall' level of symptom attribution (i.e., of ever mentioning lung cancer) from the mixed-effects logistic regression, adjusting for gender, GP experience, number of co-workers and with a random intercept for each individual GP.

Table S.3 Odds ratio, 95% CIs and p values for 'most likely' level of symptom attribution (i.e., of mentioning lung cancer as the most likely diagnosis) from the mixed-effects logistic regression with a random intercept for each individual GP.

	Overall			
**Predictor**	**OR**	**95% CI**		**P**
		**Lower**	**Upper**	
**Unadjusted**				
Vignette (COPD & General vs <u>No History & Respiratory</u>)	***67.69***	***20.47***	***223.85***	***<0.001***
Vignette (No History & General vs <u>No History & Respiratory</u>)	***73.78***	***22.13***	***245.99***	***<0.001***
Vignette (COPD & Respiratory vs <u>No History & Respiratory</u>)	*1.33*	*0.46*	*3.84*	*0.594*

Table S.4 Odds ratio, 95% CIs and p values for 'most likely' level of symptom attribution (i.e., of mentioning lung cancer as the most likely diagnosis) from the mixed-effects logistic regression, adjusting for gender, GP experience, number of co-workers and with a random intercept for each individual GP.

	**Overall**			
**Predictor**	**OR**	**95% CI**		**P**
		**Lower**	**Upper**	
**Adjusted for GP Gender, Experience and Number of Co-workers**				
Vignette (COPD & General vs No History & Respiratory)	***69.34***	***20.83***	***230.88***	***<0.001***
Vignette (No History & General vs No History & Respiratory)	***75.04***	***22.36***	***251.84***	***<0.001***
Vignette (COPD & Respiratory vs No History & Respiratory)	*1.33*	*0.46*	*3.84*	*0.596*
Gender (Male vs Female)	***2.97***	***1.12***	***7.87***	***0.028***
GP Experience (11-20 vs 1-10 years)	*2.65*	*0.68*	*10.25*	*0.159*
GP Experience (>20 vs 1-10 years)	*3.2*	*0.84*	*12.14*	*0.088*
GP Co-workers (4-5 vs Solo-3)	*0.74*	*0.15*	*3.56*	*0.704*
GP Co-workers (>5 vs Solo-3)	*1.83*	*0.44*	*7.57*	*0.407*

Note: Bold = p<0.05

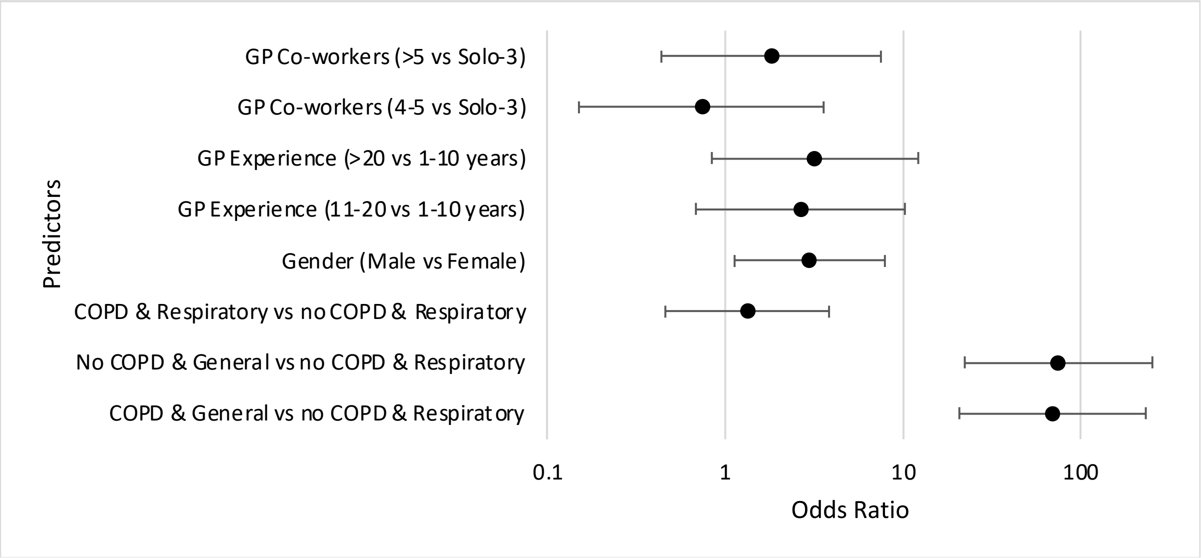

Figure S.2 Odds ratio and 95% CIs for 'most likely' level of symptom attribution (i.e., of mentioning lung cancer as the most likely diagnosis) from the mixed-effects logistic regression, adjusting for gender, GP experience, number of co-workers with a random intercept for each individual GP.

Supplementary material: Clinical decision-making on lung cancer investigations in primary care: a vignette study

Management approach analysis

Table S.5 Odds ratio, 95% CIs and p values for 'top priority' management action (i.e., selected urgent chest x-ray as the top priority action) from the mixed-effects logistic regression with a random intercept for each individual GP.

	Overall			
**Predictor**	**OR**	**95% CI**		**P**
		**Lower**	**Upper**	
**Adjusted for GP Gender, Experience, Number of Co-workers and lung cancer attribution**				
Vignette (COPD & General vs No History & Respiratory)	*0.9*	*0.46*	*1.74*	*0.744*
Vignette (No History & General vs No History & Respiratory)	*1.06*	*0.55*	*2.05*	*0.867*
Vignette (COPD & Respiratory vs No History & Respiratory)	*0.59*	*0.30*	*1.14*	*0.117*

Table S.6 Odds ratio, 95% CIs and p values for 'top priority' management action (i.e., selected urgent chest x-ray as the top priority action) from the mixed-effects logistic regression, adjusting for gender, GP experience, number of co-workers and with a random intercept for each individual GP.

	Overall			
**Predictor**	**OR**	**95% CI**		**P**
		**Lower**	**Upper**	
**Adjusted for GP Gender, Experience, Number of Co-workers and lung cancer attribution**				
Vignette (COPD & General vs No History & Respiratory)	*0.91*	*0.47*	*1.75*	*0.767*
Vignette (No History & General vs No History & Respiratory)	*1.06*	*0.55*	*2.05*	*0.865*
Vignette (COPD & Respiratory vs No History & Respiratory)	*0.59*	*0.3*	*1.15*	*0.12*
Gender (Male vs Female)	*1.23*	*0.57*	*2.68*	*0.593*
GP Experience (11-20 vs 1-10 years)	*0.67*	*0.23*	*1.97*	*0.472*
GP Experience (>20 vs 1-10 years)	*2.01*	*0.69*	*5.87*	*0.203*
GP Co-workers (4-5 vs Solo-3)	*1.56*	*0.44*	*5.53*	*0.492*
GP Co-workers (>5 vs Solo-3)	*2.03*	*0.64*	*6.43*	*0.231*

Supplementary material: Clinical decision-making on lung cancer investigations in primary care: a vignette study

Table S.7 Odds ratio, 95% CIs and p values for 'top priority' management action (i.e., selected urgent chest x-ray as the top priority action) from the mixed-effects logistic regression, adjusting for gender, GP experience, number of co-workers, 'overall' lung cancer attribution and with a random intercept for each individual GP.

	**Overall**			
**Predictor**	**OR**	**95% CI**		**P**
		**Lower**	**Upper**	
**Adjusted for GP Gender, Experience, Number of Co-workers and lung cancer attribution**				
Vignette (COPD & General vs No History & Respiratory)	*0.76*	*0.38*	*1.51*	*0.433*
Vignette (No History & General vs No History & Respiratory)	*0.9*	*0.46*	*1.78*	*0.769*
Vignette (COPD & Respiratory vs No History & Respiratory)	*0.59*	*0.3*	*1.15*	*0.121*
Gender (Male vs Female)	*1.16*	*0.53*	*2.53*	*0.717*
GP Experience (11-20 vs 1-10 years)	*0.58*	*0.2*	*1.73*	*0.329*
GP Experience (>20 vs 1-10 years)	*1.73*	*0.58*	*5.11*	*0.325*
GP Co-workers (4-5 vs Solo-3)	*1.51*	*0.42*	*5.42*	*0.53*
GP Co-workers (>5 vs Solo-3)	*1.85*	*0.58*	*5.96*	*0.301*
Overall lung cancer attribution (Lung cancer vs Not)	***2.51***	***1.24***	***5.09***	***0.011***

Note: Bold = p<0.05

Table S.8 Odds ratio, 95% CIs and p values for 'top priority' management action (i.e., selected urgent chest x-ray as the top priority action) from the mixed-effects logistic regression, adjusting for gender, GP experience, number of co-workers, 'most likely' lung cancer attribution and with a random intercept for each individual GP.

	**Overall**			
**Predictor**	**OR**	**95% CI**		**P**
		**Lower**	**Upper**	
**Adjusted for GP Gender, Experience, Number of Co-workers and lung cancer attribution**				
Vignette (COPD & General vs No History & Respiratory)	*0.51*	*0.23*	*1.10*	*0.085*
Vignette (No History & General vs No History & Respiratory)	*0.59*	*0.29*	*1.12*	*0.178*
Vignette (COPD & Respiratory vs No History & Respiratory)	*0.57*	*0.29*	*1.12*	*0.105*
Gender (Male vs Female)	*1.09*	*0.49*	*2.39*	*0.838*
GP Experience (11-20 vs 1-10 years)	*0.60*	*0.20*	*1.77*	*0.351*
GP Experience (>20 vs 1-10 years)	*1.79*	*0.60*	*5.29*	*0.294*
GP Co-workers (4-5 vs Solo-3)	*1.63*	*0.45*	*5.87*	*0.458*
GP Co-workers (>5 vs Solo-3)	*1.92*	*0.59*	*6.19*	*0.276*
'Most likely' lung cancer attribution (Lung cancer vs Not)	***3.01***	***1.47***	***6.16***	***0.003***

Note: Bold = p<0.05

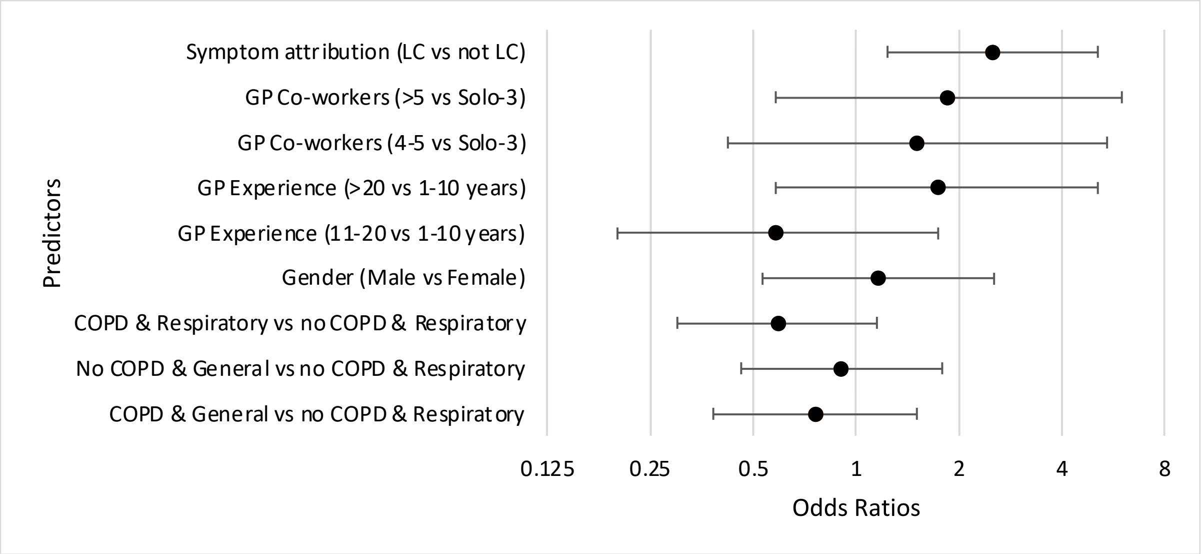

Figure S.3. Odds ratio and 95% CIs for 'top priority' management action (i.e., selected urgent chest x-ray as the top priority action) from the mixed-effects logistic regression, adjusting for gender, GP experience, number of co-workers, lung cancer attribution with a random intercept for each individual GP.

Supplementary material: Clinical decision-making on lung cancer investigations in primary care: a vignette study

Table S.9 Frequency of 'top priority' management approach by 'most likely' level symptom attribution (i.e., of mentioning lung cancer as the most likely diagnosis) clustered by symptom type.

Top priority management approach		Most likely symptom attribution				
		**Lung cancer n (%)**	**COPD n (%)**	**Resp cond. n (%)**	**Cardiac cond. n (%)**	**Other cond. n (%)**
**dyspnoea + persistent cough**	Refer to A&E	2 (9)	0	1 (1)	0	1 (4)
	Urgent Chest x-ray	15 (68)	30 (42)	41 (58)	7 (41)	12 (44)
	Non-urgent Chest x-ray	0	1 (1)	3 (4)	0	3 (11)
	Referral to respiratory specialist	2 (9)	0	1 (1)	0	0
	Refer to COPD Nurse, Pulmonary rehabilitation programme or Spirometry	0	1 (1)	12 (17)	0	0
	Blood tests (e.g. FBC panel, U&E, LFT, CRP or BNP)	2 (9)	5 (7)	7 (10)	8 (47)	4 (15)
	Sputum culture	0	3 (4)	1 (1)	0	1 (4)
	CT Scan	1 (5)	0	3 (4)	0	0
	ECG	0	0	0	2 (12)	0
	Prescribe or change medications and book an appointment for review within a few days	0	31 (43)	2 (3)	0	6 (22)
	Monitor symptoms (e.g. Ask patient to keep an eye on the symptoms and to return if they worsen or change)	0	1 (1)	0	0	0
	Total (N)	22	72	71	17	27
**fatigue + weight loss**	Refer To A&E	3 (2)	0	2 (7)	0	0
	Urgent Chest x-ray	82 (62)	4 (57)	16 (55)	1 (25)	13 (32)
	Non-Urgent Chest x-ray	3 (2)	0	0	0	0
	Referral to respiratory specialist	16 (12)	0	0	0	2 (5)
	Refer to COPD Nurse or Spirometry	1 (1)	0	2 (7)	0	0
	Gastroenterologist referral	0	0	0	0	2 (5)
	Blood tests (e.g. FBC panel, U&E, LFT, CRP)	14 (11)	0	5 (17)	2 (50)	21 (51)
	CT Scan	13 (10)	1 (14)	3 (10)	1 (25)	1 (2)
	ECG	0	0	1 (4)	0	0
	Prescribe or change medications and book an appointment for review within a few days	0	2 (29)	0	0	2 (5)
	Total (N)	132	7	29	4	41
