# Supplementary material for: Clinical decision-making on lung cancer investigations in primary care: a vignette study
Source: BMJ Open. 2024 Aug 21;14(8):e082495. doi: 10.1136/bmjopen-2023-082495 (PMC11340710; doi:10.1136/bmjopen-2023-082495)
Supplement: Supplementary file 2 [file bmjopen-2023-082495.reviewer_comments.pdf]

PEER REVIEW HISTORY

BMJ Open publishes all reviews undertaken for accepted manuscripts. Reviewers are asked to complete a checklist review form and are provided with free text boxes to elaborate on their assessment. These free text comments are reproduced below.

ARTICLE DETAILS

**TITLE (PROVISIONAL)**	Clinical decision-making on lung cancer investigations in primary care: a vignette study
**AUTHORS**	Mitchinson, Lucy; von Wagner, Christian; Blyth, Alexandra; Shah, Heer; Rafiq, Meena; Merriel, Samuel; Barclay, Matthew; Lyratzopoulos, Georgios; Hamilton, Willie; Abel, Gary; Renzi, Cristina

VERSION 1 - REVIEW

**REVIEWER NAME**	***Harris, Michael***
**REVIEWER AFFILIATION**	University of Bath, Department for Health
**REVIEWER CONFLICT OF INTEREST**	None
**DATE REVIEW RETURNED**	25-Jan-2024

**GENERAL COMMENTS**	Early diagnosis of lung cancer can reduce the poor survival rate in patients with the condition, and research that explores GPs' decision making in patients that could have lung cancer is welcome.<br>'Chronic Obstructive Pulmonary Disease (COPD) affects 2% of the UK population and is associated with twice the risk of lung cancer.' A reader may assume from this statement that it is because lung cancer and COPD have a common aetiology (smoking). The authors need to make it clear that COPD is an independent risk factor for lung carcinoma.<br>'We could expect GPs to refer patients with COPD for urgent x-ray more readily due to the increased cancer risk' – we could only expect this if the presence of COPD as an independent risk factor for lung carcinoma were well known by GPs.<br>The second research question is unclear. Is it asking how a patient's COPD-status influences a GP's decision to refer the patient for an urgent chest x-ray? Or is it asking how a patient's COPD-status influences a GP's decision to refer the patient for an urgent chest x-ray when the patient has respiratory symptoms, compared with when the patient has general symptoms? The study is described as a 2x2 mixed factorial design, so presumably three questions could be posed and stated here: How does a patient's COPD-status influence a GP's decision to refer the patient for an urgent chest x-ray? How does whether a patient has respiratory or general symptoms influence a GP's decision to refer the patient for an urgent chest x-ray? Is there an interaction between the two?<br>'Participants were UK GPs.' How were these 109 GPs selected and recruited?<br>I recommend having a separate sub-heading, early in the Methods section, for 'Outcome measures' – what are the outcome measures for each of the research questions?<br>Although the research questions suggest that the outcome measures should relate to 'lung cancer' and 'not lung cancer', and 'urgent CXR' and 'not urgent CXR', multiple other factors are

	presented, tabulated, highlighted and discussed. The results and the discussion should be limited to those relating to the outcome measures (retaining the information about the demographics, of course). This needs to be reflected in the Abstract.<br>Strengths and limitations. There needs to be a discussion about whether the way the GPs were sampled may have caused bias.

**REVIEWER NAME**	***Hollmen, Maria***
**REVIEWER AFFILIATION**	University of Helsinki, Heart and Lung center Department of Pulmonary medicine
**REVIEWER CONFLICT OF INTEREST**	None
**DATE REVIEW RETURNED**	15-Mar-2024

**GENERAL COMMENTS**	Dr Mitchinson et al. Have done a very nice and interesting study on GP clinical decision making in relation to lung cancer. This is a very important topic that has not been studied enough.<br><br>Altogether 109 GP:s completed 422 vignettes online.<br><br>The vignettes were clear and similar enough for the purposes. In my opinion, 3 weeks is quite a short time span so that the most probable diagnosis with respiratory symptoms is not lung cancer. I, as a pulmonologist think that a 3 week time span is more suited for COPD, asthma or infection. Also, lung cancer but in second line. If it was 3 months this would totally change the situation. This should be added as a limitation to the study. It is stated in the limitations of the study that mentioning the smoking status automatically increases the likelihood for considering lung cancer. Yes, this is true, but I don't think it is a limitations as actually it reflects the real life situation well – the patients are asked about smoking history. Additionally, I think it would be really important to focus on the issue that some kind of control/re-visit was arranged for the patient after for example adjusting medication. In real life, this often is left undone but is immensely important.<br><br>I am not quite sure how the GP's were selected based on the methods section. Also, it would need to be stated whether the GP´s (their experience, size of the practice etc.) represent well the GP´s in the UK in general. This needs to be added to the manuscript. It is difficult for me to assess whether 109 GP's is a good amount or not as I am not familiar with the GP practices in the UK so this would need to be discussed in the discussion section. To clarify I think it is enough for this study, but it would need to be discussed. What was a target number for GP's to be recruited and was this achieved?<br><br>The introduction is quite approximate, and I encourage the authors to broaden it.<br><br>About the statistics:<br>A mixed-effects logistic regression was used but I could not find a statement that the assumptions were tested and met. This test is very sensitive to outliers and assumes that the relationship between the natural log of these probabilities (when expressed as odds) and your predictor variable is linear. I could not find a statement about outliers – were there any? And if yes what was done to them? How about the multicollinearity? Was this tested? And it also needs to be

	shown. In summary, the assumptions need to be tested and shown that they are fulfilled so that it is ok to use this test.<br><br>You state that ‘univariable regression analyses were used to compare frequency of suggested diagnosis within each symptom by COPD status’ which regression analyses were used? Logistic? Something else? This needs to be cleared.<br><br>On whole I think that the discussion is quite superficial and needs to be significantly broadened. It additionally wasn’t easy to understand. I read it several times and still I was left thinking what do you think would be the optimal result? As I already said before, I think that in case of respiratory symptoms for 3 weeks it is not realistic to expect that the most likely diagnosis that comes to mind is cancer when the patient has COPD. This would need to be discussed. Also, what do you suggest that should be done to improve this situation, instead of just recommend more studies on this topic. The comparison to previous literature was shallow and would need to be strengthened.<br><br>Minor comments:<br><br>Page 4 line 14 I suggest adding a reference or 2 showing that this has also been shown in international studies<br><br>Page 4 lines 31-35, is there a reference for this? Or is it just an assumption?<br>Page 5 line 39 what was considered too short? And how many were excluded?<br><br>The tables and figures need to be a more refined. Now they are too unfinished and hard to read. For example, Table 1 says experience (years) and still years is written after the number in each row. Some words are written in capital letters such as Spirometry and some not. There is no consistency in these. The row total should be positioned differently. It is important to write footnotes to tables where all abbreviations are clarified (for example A&E, lab tests etc). Also, the groups and parameters should be named so that the reader easily understand which groups are meant in each row and so on. So please re-do the tables and figures carefully.<br><br>I think that the conclusion especially in the abstract is too vague and needs to be more clear and precise.<br><br>In summary, this is a very nice study but the manuscript is a bit unfinished.

**REVIEWER NAME**	***Benson, Ryzen***
**REVIEWER AFFILIATION**	UCSF, Department of Radiation Oncology
**REVIEWER CONFLICT OF INTEREST**	Not related to this research, but in 2023 I served as an informatics consultant for a public health company.
**DATE REVIEW RETURNED**	19-Mar-2024

GENERAL COMMENTS	I would like to thank the authors for allowing me to review their study evaluating clinical decision-making for lung cancer. I found the use of vignettes coupled with the factorial design to be very interesting and I enjoyed reading about your research. Please see my comments below:<br><br>1) In the first paragraph, the authors mention how late diagnosis of cancer greatly impacts survival. Can you please add numbers to greater emphasize the impact? Also, I think the first two paragraphs ("Chronic Obstructive ...", and "Clinical decisions...") would flow better if combined into one single paragraph.<br><br>2) In the results, the 'Symptom Attribution' and 'Management Approaches' sections were difficult to follow when jumping back and forth between multiple-factor comparisons. I felt that I was only able to truly understand the results when looking at the tables.<br><br>Can the authors please explicitly state which factors are being compared in some sort of systematic approach (e.g., Top 3 symptom attributions for COPD and worsening resp., top 3 symptom attributions for No COPD and worsening resp.)<br><br>3) In tables 2, 3 and 4, symptom attribution is being assessed for either worsening respiratory symptoms (dyspnea + persistent cough) or worsening general symptoms (fatigue + weight loss) in COPD vs no COPD. However, the symptom names are only on the table and not the name of the factor. It would be helpful if that cell said something like Worsening respiratory symptoms (dyspnea + persistent cough) and Worsening general symptoms (fatigue + weight loss).<br><br>4) The discussion seems too short. I still don't feel like I understand the big takeaway in the discussion section, but it all makes more sense in the following subsections (e.g., comparison with prior literature, implications for research and/or practice).<br><br>I think it would be much easier to follow along if the authors had one discussion section that compares and contrasts their work with prior literature and describes the conclusions/implications of the work. That way these ideas aren't too split up by the subheadings.<br><br>5) In the symptom attribution subsection in results, this sentence: "Examining worsening general symptoms, lung cancer was indicated by GPs as the 'most likely' cause in 65% of patients with COPD and 63% without (Table 2)." These numbers do not match the proportions listed in table 2.

VERSION 1 – AUTHOR RESPONSE

Reviewer 1:

2. 'Chronic Obstructive Pulmonary Disease (COPD) affects 2% of the UK population and is associated with twice the risk of lung cancer.' A reader may assume from this statement that it is because lung cancer and COPD have a common aetiology (smoking). The authors need to make it clear that COPD is an independent risk factor for lung carcinoma.

- *We have now made it clear that the COPD is an independent risk factor for lung cancer with the following sentence: "Chronic Obstructive Pulmonary Disease (COPD) affects 2% of the UK population and is an independent risk factor associated with twice the risk of lung cancer".*

3. 'We could expect GPs to refer patients with COPD for urgent x-ray more readily due to the increased cancer risk' – we could only expect this if the presence of COPD as an independent risk factor for lung carcinoma were well known by GPs.

- *We have edited this sentence to now read: "GPs may be aware of the link between COPD and lung cancer and refer patients with COPD for urgent x-ray more readily due to the increased cancer risk; however, the alternative explanation mechanism suggests some GPs may instead attribute the symptoms to COPD and choose to manage the condition rather than refer for an x-ray".*

4. The second research question is unclear. Is it asking how a patient's COPD-status influences a GP's decision to refer the patient for an urgent chest x-ray? Or is it asking how a patient's COPD-status influences a GP's decision to refer the patient for an urgent chest x-ray when the patient has respiratory symptoms, compared with when the patient has general symptoms? The study is described as a 2x2 mixed factorial design, so presumably three questions could be posed and stated here: How does a patient's COPD-status influence a GP's decision to refer the patient for an urgent chest x-ray? How does whether a patient has respiratory or general symptoms influence a GP's decision to refer the patient for an urgent chest x-ray? Is there an interaction between the two?

- *Thank you for highlighting this. We agree that our research questions could be presented in a clearer way.*

- *We now present three research questions which focus on 1) the effect of COPD on symptom attribution to lung cancer, 2) the effect of COPD on referral to urgent chest x-ray, and 3) the effect of COPD in combination with symptom type on the decision to refer for urgent chest x-ray. We have also restructured the presentation of our results, dividing the work into the primary and secondary outcomes and by research question. We feel that this division makes the methods and results easier to follow and interpret.*

5. 'Participants were UK GPs.' How were these 109 GPs selected and recruited?

- *We have now expanded the description of recruitment to say the following: "Participants were UK GPs who were registered as responders on the Dynata survey platform (www.dynata.com). Participants who matched the criteria of; 1) being based in the UK and 2) being qualified GPs, were recruited in May and June of 2022 via a Dynata advertisement. No additional inclusion or exclusion criteria were applied. GPs were compensated £38.50 for completing the survey. Participants whose survey completion times were deemed too short were removed for quality assurance purposes (e.g. < 30 seconds)."*

- *We have also included information on national GP demographics (e.g. gender divide and practice size) in the results section. We note that our sample included a greater proportion of GPs working in larger practices and fewer in single GP practices than occurs nationwide.*

6. I recommend having a separate sub-heading, early in the Methods section, for ‘Outcome measures’ – what are the outcome measures for each of the research questions?

- *An outcome measures section has now been added to the methods following the section on Study Design. In this section, we restate the research question and describe how the data was gathered to measure symptom attribution and management approaches. The section reads as follows:*

Outcome measures

a. Symptom attribution

To explore how a patient’s COPD-status influences a GP’s attribution of worsening respiratory or general symptoms to lung cancer (research question 1), GPs were asked: ‘What do you think are the three most likely diagnoses, starting from the most likely to the least likely?’ after reading each vignette. The free-text responses were analysed and coded into categories developed during content analysis (e.g., ‘Lung Cancer’ or ‘Respiratory Infections’) to form the measure of ‘symptom attribution’.

To explore the primary attribution outcome (e.g. attribution of symptoms to lung cancer) the responses were dichotomised to ‘lung cancer’ or ‘not lung cancer’ for analyses. To explore the secondary attribution outcome (e.g. attribution of symptoms to alternative diagnoses), the initial attribution categories were used.

b. Management approaches

To explore whether a patient’s COPD-status influences a GP’s decision to refer the patient for an urgent chest x-ray (research question 2), and whether this changed when presenting in combination with respiratory vs. general symptoms (research question 3), participants were asked to choose up to four management approaches from a list of 20 pre-coded options. The approaches were ranked by priority, with ‘1’ being “highest priority” and ‘4’ being “lowest priority” to form the measure of management approach.

To explore the primary management outcome (e.g. referral for urgent chest x-ray) responses were dichotomised to ‘urgent chest x-ray’ or ‘not urgent chest x-ray’. Only the highest priority approach (out of 4 possible responses) was considered for this analysis, as delays might occur if urgent chest x-ray was not the top priority. To explore the secondary management outcome (e.g. selection of other management approaches by GPs) all management approach options were used in analysis.

a. Although the research questions suggest that the outcome measures should relate to ‘lung cancer’ and ‘not lung cancer’, and ‘urgent CXR’ and ‘not urgent CXR’, multiple other factors are presented, tabulated, highlighted and discussed. The results and the discussion should be limited to those relating to the outcome measures (retaining the information about the demographics, of course). This needs to be reflected in the Abstract.

- *Thank you for raising this. Our primary outcomes of interest are attribution of symptoms to lung cancer and decision-making on referral for urgent chest x-ray. However, we collected additional data on alternative diagnoses and management approaches suggested by the GPs. While we note that urgent chest x-ray referral did not vary significantly by vignette, the additional data sheds light on other potential mechanisms for delay in cancer diagnosis for patients with COPD.*

- *To make our reporting clearer we have added a statement of our primary and secondary outcomes early in the paper. In our results section we now present the primary and secondary outcomes separately.*

- *We have adapted the abstract to report on the primary outcomes only due to word limits.*

b. Strengths and limitations. There needs to be a discussion about whether the way the GPs were sampled may have caused bias.

- *We have now included a discussion on the potential limitations of our sampling approach, namely a potential lack of representation and generalisability. The section reads 'Lastly, GPs were recruited via an online survey platform with limited inclusion criteria. While this allowed for wide participation of UK GPs, our sample was not directly representative of the UK. We had a greater proportion of GPs who worked in larger practices. There is a potential that GPs from larger practices experience more patients with lung cancer or have better systems in place for lung cancer referral.'*

Reviewer 2:

c. The vignettes were clear and similar enough for the purposes. In my opinion, 3 weeks is quite a short time span so that the most probable diagnosis with respiratory symptoms is not lung cancer. I, as a pulmonologist think that a 3 week time span is more suited for COPD, asthma or infection. Also, lung cancer but in second line. If it was 3 months this would totally change the situation. This should be added as a limitation to the study. It is stated in the limitations of the study that mentioning the smoking status automatically increases the likelihood for considering lung cancer. Yes, this is true, but I don't think it is a limitations as actually it reflects the real life situation well – the patients are asked about smoking history. Additionally, I think it would be really important to focus on the issue that some kind of control/re-visit was arranged for the patient after for example adjusting medication. In real life, this often is left undone but is immensely important.

- *Thank you for your reflections on this element. The three-week time period was chosen as NICE guidelines and public health advice (such as the Be Clear campaign) recommend that symptoms which persist for 3-weeks are investigated further. We understand that other respiratory conditions may be likely causes of such symptoms and tried to account for this by investigating the top three most likely diagnoses. We feel that the persistent nature of these symptoms would be sufficient to increase suspicion of cancer and warrant further investigation as per NICE guidelines. We have explored this further within the discussion to ensure we address these concerns.*

- *We agree that a revisit appointment or some form of monitoring would be an important outcome to consider in future vignette studies, particularly when considering a longer period of symptoms (such as 3-months compared to 3-weeks). We will keep this in mind for our future studies and have included this in the discussion as a suggestion for future research.*

d. I am not quite sure how the GP's were selected based on the methods section. Also, it would need to be stated whether the GP´s (their experience, size of the practice etc.) represent well the GP´s in the UK in general. This needs to be added to the manuscript. It is difficult for me to assess whether 109 GP's is a good amount or not as I am not familiar with the GP practices in the UK so this would need to be discussed in the discussion section. To clarify I think it is enough for this study, but it would need to be discussed. What was a target number for GP's to be recruited and was this achieved?

- We have now expanded the section of GP recruitment by adding additional details. The target number of GPs was recruited, and the target number of vignettes were completed. We have provided the gender and practice size breakdown of the UK so our sample can be compared (see results paragraph 1). We have noted the greater representation of larger GP practices and the possible implications in terms of generalisability have been mentioned in the limitations section.

e. The introduction is quite approximate, and I encourage the authors to broaden it.

- We have now developed our introduction to include greater scene setting by explaining the conditions and supporting theories more deeply. We have also provided more details on the impact of late diagnosis on survival. We feel the introduction is now more fully developed and provides a clearer entry to the paper.

f. A mixed-effects logistic regression was used but I could not find a statement that the assumptions were tested and met. This test is very sensitive to outliers and assumes that the relationship between the natural log of these probabilities (when expressed as odds) and your predictor variable is linear. I could not find a statement about outliers – were there any? And if yes what was done to them? How about the multicollinearity? Was this tested? And it also needs to be shown. In summary, the assumptions need to be tested and shown that they are fulfilled so that it is ok to use this test.

- Thank you for this comment. Our analytical approach was guided by the study statistician. As the outcome data for this model was coded to be binary (0s or 1s) we are unsure what the reviewer means by outliers. We did not search for 'outliers' or exclude observations, as this in general would be expected to introduce bias. It should be noted that there was potential for data to be excluded for purposes of data quality (i.e., odd covariate values).

We did not formally search for multicollinearity as this generally is not viewed as a problem for vignette research. The finite standard errors are also not suggestive of multicollinearity. We are confident that the analytical approach is valid without carrying out further tests.

g. You state that 'univariable regression analyses were used to compare frequency of suggested diagnosis within each symptom by COPD status' which regression analyses were used? Logistic? Something else? This needs to be cleared.
- This has now been adapted and is stated as logistic univariate regression analyses throughout the methods section.

h. On whole I think that the discussion is quite superficial and needs to be significantly broadened. It additionally wasn't easy to understand. I read it several times and still I was left thinking what do you think would be the optimal result? As I already said before, I think that in case of respiratory symptoms for 3 weeks it is not realistic to expect that the most likely diagnosis that comes to mind is cancer when the patient has COPD. This would need to be discussed. Also, what do you suggest that should be done to improve this situation, instead of just recommend more studies on this topic. The comparison to previous literature was shallow and would need to be strengthened.

- *Thank you for your feedback on the Discussion. Whilst we agree that not all patients with symptoms lasting three weeks will have significant underlying pathology, NICE guidance for GPs and recent NHS England 'Be Clear on Cancer' campaigns both highlight that the presence of persistent respiratory symptoms (for three weeks or more) necessitates contacting primary care for further assessment and should indicate sufficient concern for GP investigation. We attempted to account for the other respiratory conditions which may be likely causes of such symptoms by including exploring both the 'most likely' and 'top three' likely suggested diagnoses.*

- *We have clarified our recommendations for practice within the Discussion. We recommend increasing clinician awareness of the increased lung cancer risk for patients with COPD and encourage consideration of further investigation in patients with COPD who have persistent and worsening symptoms, or do not respond to initial drug treatments. This could be supported by the use of decision support tools which incorporate comorbidities (such as COPD) and additional electronic health record data to identify patients who are at a higher risk of underlying lung cancer and should be prioritised for urgent referrals. Further research identifying the clinical features associated with underlying lung cancer in patients with COPD could also inform updates to NICE guidelines.*

- *We have significantly reworked this section to make the key findings clear and place the findings in the context of emerging evidence for the effect of comorbidities on lung cancer diagnosis.*

i. Minor comments:

Page 4 line 14 I suggest adding a reference or 2 showing that this has also been shown in international studies.

Page 4 lines 31-35, is there a reference for this? Or is it just an assumption?

Page 5 line 39 what was considered too short? And how many were excluded?

- *Thank you for highlighting these aspects. We have provided additional references from international studies and detail on the exclusion criteria.*

j. The tables and figures need to be a more refined. Now they are too unfinished and hard to read. For example, Table 1 says experience (years) and still years is written after the number in each row. Some words are written in capital letters such as Spirometry and some not. There is no consistency in these. The row total should be positioned differently. It is important to write footnotes to tables where all abbreviations are clarified (for example A&E, lab tests etc). Also, the groups and parameters should be named so that the reader easily understand which groups are meant in each row and so on. So please re-do the tables and figures carefully.

- *Thank you for comments on the tables and figures. We have adapted the tables to fit with the guidance outlined on the 'Improving your tables and figures' videos available on BMJ Open.*

k. I think that the conclusion especially in the abstract is too vague and needs to be more clear and precise.

- *Thank you for the suggestion. The conclusion in the abstract has been refined.*

Reviewer 3:

l. In the first paragraph, the authors mention how late diagnosis of cancer greatly impacts survival. Can you please add numbers to greater emphasize the impact? Also, I think the first two paragraphs ("Chronic Obstructive ...", and "Clinical decisions...") would flow better if combined into one single paragraph.

- *We agree that providing values clarifies our message. We have now included values from a recent paper published in Thorax in 2022.*

- *We have also now joined the two opening paragraphs to improve flow.*

m. In the results, the 'Symptom Attribution' and 'Management Approaches' sections were difficult to follow when jumping back and forth between multiple-factor comparisons. I felt that I was only able to truly understand the results when looking at the tables. Can the authors please explicitly state which factors are being compared in some sort of systematic approach (e.g., Top 3 symptom attributions for COPD and worsening resp., top 3 symptom attributions for No COPD and worsening resp.)

- *We have now divided our findings into primary and secondary outcomes, and provided separate sections for the data which directly addresses the research questions. We feel that this approach has made the results easier to follow.*

- *We have standardised the language to make the results easier to interpret. We now state the symptom type and COPD-status in full during comparisons.*

n. In tables 2, 3 and 4, symptom attribution is being assessed for either worsening respiratory symptoms (dyspnea + persistent cough) or worsening general symptoms (fatigue + weight loss) in COPD vs no COPD. However, the symptom names are only on the table and not the name of the factor. It would be helpful if that cell said something like Worsening respiratory symptoms (dyspnea + persistent cough) and Worsening general symptoms (fatigue + weight loss).

- *Thank you for this suggestion. We have now adjusted the table titles to Worsening general symptoms (fatigue + weight loss) and Worsening respiratory symptoms (dyspnoea + persistent cough).*

o. The discussion seems too short. I still don't feel like I understand the big takeaway in the discussion section, but it all makes more sense in the following subsections (e.g., comparison with prior literature, implications for research and/or practice). I think it would be much easier to follow along if the authors had one discussion section that compares and contrasts their work with prior literature and describes the conclusions/implications of the work. That way these ideas aren't too split up by the subheadings.

- *Thank you for the feedback. Please see our response to reviewer 2 (point 14).*

p. In the symptom attribution subsection in results, this sentence: "Examining worsening general symptoms, lung cancer was indicated by GPs as the 'most likely' cause in 65% of patients with COPD and 63% without (Table 2)." These numbers do not match the proportions listed in table 2.

- *Thank you for spotting this. The values have now been corrected to 61% of patients with COPD and 63% without.*

VERSION 2 – REVIEW

**REVIEWER NAME**	***Harris, Michael***
**REVIEWER AFFILIATION**	University of Bath, Department for Health
**REVIEWER CONFLICT OF INTEREST**	No competing interests
**DATE REVIEW RETURNED**	13-Jul-2024

**GENERAL COMMENTS**	The authors have made substantial changes to the paper that have addressed the criticisms and recommendations that I listed in my previous review.

**REVIEWER NAME**	***Hollmen, Maria***
**REVIEWER AFFILIATION**	University of Helsinki, Heart and Lung center Department of Pulmonary medicine
**REVIEWER CONFLICT OF INTEREST**	None
**DATE REVIEW RETURNED**	19-Jul-2024

**GENERAL COMMENTS**	The authors have taken every reviewer's comments seriously and changed the manuscript considerabely. I am pleased with this and my previous questions have been answered. I don't have any further comments or questions.
